# Modules or Mean-Fields?

**DOI:** 10.3390/e22050552

**Published:** 2020-05-14

**Authors:** Thomas Parr, Noor Sajid, Karl J. Friston

**Affiliations:** Wellcome Centre for Human Neuroimaging (UCL), London WC1N 3AR, UK; noor.sajid.18@ucl.ac.uk (N.S.); k.friston@ucl.ac.uk (K.J.F.)

**Keywords:** stochastic dynamics, modularity, density dynamics, message passing, Bayesian mechanics

## Abstract

The segregation of neural processing into distinct streams has been interpreted by some as evidence in favour of a modular view of brain function. This implies a set of specialised ‘modules’, each of which performs a specific kind of computation in isolation of other brain systems, before sharing the result of this operation with other modules. In light of a modern understanding of stochastic non-equilibrium systems, like the brain, a simpler and more parsimonious explanation presents itself. Formulating the evolution of a non-equilibrium steady state system in terms of its density dynamics reveals that such systems appear on average to perform a gradient ascent on their steady state density. If this steady state implies a sufficiently sparse conditional independency structure, this endorses a mean-field dynamical formulation. This decomposes the density over all states in a system into the product of marginal probabilities for those states. This factorisation lends the system a modular appearance, in the sense that we can interpret the dynamics of each factor independently. However, the argument here is that it is *factorisation*, as opposed to *modularisation*, that gives rise to the functional anatomy of the brain or, indeed, any sentient system. In the following, we briefly overview mean-field theory and its applications to stochastic dynamical systems. We then unpack the consequences of this factorisation through simple numerical simulations and highlight the implications for neuronal message passing and the computational architecture of sentience.

## 1. Introduction

Attempts to understand neuroanatomical and psychological organisation have often appealed to the notion of a ‘module’ [1,2,3,4,5]. The basic idea is that cognition depends upon a set of specialised modules that operate (almost) independently of one another. Each module is thought to receive a specialised form of input—often a specific sensory modality—and provides a low dimensional output to other modules. It is easy to see the appeal of this kind of formulation. Just as we think of the heart as an organ to pump blood, the kidneys to filter it, and the lungs to oxygenate it, the modular perspective on cognitive function lets us (literally) organize the brain into constituent organs that each play their own role in processing information. The occipital cortices are ‘for’ processing visual data, the ventral visual stream ‘for’ identifying the thing that caused these data and the dorsal stream ‘for’ locating these causes. Often, this teleological perspective is motivated in terms of evolutionary psychology [6]. Pragmatically, this suggests an approach to evaluating cognitive function. If we can think of the brain in terms of functionally specialised modules, it should be possible to design experiments and cognitive tests that interrogate these, independently of one another. In this paper, we argue that the emergence of a modular architecture is more simply expressed in terms of factorisation. This perspective arises from an approach developed in statistical physics called mean-field theory [7,8]. The basic idea is that a probability distribution over the components of a system may be approximated by the product of the distributions for each component (or groups of components) of that system. This treatment assumes we can treat parts of the system as operating independently to other parts, just as modules are treated as independent of one another. In addition to neurobiology [9,10], applications of mean-field theory are broad, and have been used to find tractable solutions to problems in fields as diverse as statistics [11], soft-matter physics [12], epidemiology [13], game theory [14], and financial modelling [15].

Section 2 provides a review of mean-field theory, the problem it was developed to solve, and the form of the solution. Interestingly, this solution does not involve complete independence of each factor. Instead it ensures the components of a system depend upon one another via their mean-fields—so-called because only the average values of other components matter. Section 3 takes the concept of mean-field theory and places it in a dynamical context. We set out the density dynamics of mean-field systems at (non-equilibrium) steady state. Doing so reveals that each factorised component appears to undergird its own steady state density. However, the steady state density of each factor depends upon the mean field of other factors. Section 4 introduces some minimal simulations that aim to build an intuition as to how this works in practice. These numerical analyses are designed to illustrate the ideas introduced in earlier sections as simply as possible. Here, we see a simple form of functional (modular) specialisation, and the emergence of a separation of timescales that is characteristic of hierarchical neuronal dynamics. Section 5 highlights the link between mean-field formalism, inference, and the message passing between populations of neurons. This rests on the fact that the simplest tractable way to make inferences about the causes of (sensory) data is to use a mean field approximation that underwrites a form of variational or approximate Bayesian inference. In short, we can study the properties of stochastic dynamical systems—like the brain—through mean-field assumptions. This should not be interpreted as a model of the brain—rather it is an approach to understanding stochastic systems with sparse dependency structures, of which the brain is a paradigmatic example. We suggest that accounts of cognitive function in terms of modular architectures rest upon an intuitive application of mean-field theory. Making this explicit provides a useful perspective on brain function and lets us exploit established tools from stochastic physics. We start with an overview of these tools.

## 2. Mean-Field Theory

The origins of mean-field theory are in physics [7,8]. They were invoked to study systems described by a Gibbs’ measure. This is an expression of the statistical properties of a system that says that the probability density of a system being in a particular state *x* decreases as the energy associated with that state increases. In other words, the higher the total energy of the system in each configuration, the lower the probability of that configuration. Turing this on its head, energy may be thought of as a measure of the improbability of a configuration. For reasons that will be clearer later, we are interested in systems with a second random variable that takes the value *y*. This is a parameter can change the shape of the energy landscape for *x.* In the context of the neurosciences, *x* could indicate (log) neuronal firing rates with *y* indicating sensory stimulation. Through Bayes’ theorem, we can interpret the variables in this system in terms of joint, conditional, and marginal probability densities:

(1) $$\begin{array}{lll} p\left(x|y\right) & = & \frac{1}{Z(y)}e^{-\beta H(x,y)}\\ Z(y) & ≜ & \int_{-\infty }^{\infty }e^{-\beta H(x,y)}dx\\ & \Rightarrow & \ln p(x|y)=-\beta H(x,y)-\ln Z(y)\\ & \Rightarrow & \left\{\begin{array}{l} \ln p(x,y)=-\beta H(x,y)\\ \ln p(y)=\ln Z(y) \end{array}\right. \end{array}$$

The total energy of the system is given by the Hamiltonian (H). For classical dynamical systems, this is a scalar function. For quantum dynamical systems, this is a linear operator whose eigenvalues are interpretable as energies. Equation (1) is more general than it appears at a first glance. While the expression in the first line may seem restrictive, the Gibbs’ form in the first equality of Equation (1) can be used to express any exponential family probability distribution by choosing different forms for the Hamiltonian. Some common examples are given in Table 1. The integral in the second equality must be replaced by a sum when the support of the distribution is categorical. The *β* parameter is sometimes referred to as an ‘inverse temperature’ parameter, as it is inversely proportional to the temperature of a physical system. This determines how ‘peaky’ the distribution is, with high *β* concentrating probability mass on a small region of space, and low *β* leading to a more even distribution of probability mass.

The denominator—or normalising constant—(*Z*) of the first line of Equation (1) is an important quantity in thermodynamics called a partition function. This is closely related to another quantity called Helmholtz free energy [17,18]:

(2) $$\begin{array}{lll} F(y) & ≜ & -\frac{1}{\beta }\ln Z(y)\\ & = & \frac{1}{\beta }\ln p(x|y)+H(x,y)\\ & = & E_{p(x|y)}\left[\frac{1}{\beta }\ln p(x|y)+H(x,y)\right]\\ & = & U(x,y)-TS(x,y)\\ U(x,y) & ≜ & E_{p(x|y)}\left[H(x,y)\right]\\ S(x,y) & ≜ & -\frac{1}{\beta T}E_{p(x|y)}\left[\ln p(x|y)\right] \end{array}$$

Here, E indicates an expectation (i.e., average), *U* is the internal energy of the system, *T* is its temperature, and *S* is its entropy. The third equality rests upon the fact that the Helmholtz free energy does not depend upon *x*, so:

(3) $$\begin{array}{lll} E_{p(x|y)}\left[F(y)\right] & = & F(y)\\ & \Rightarrow & E_{p(x|y)}\left[\frac{1}{\beta }\ln p(x|y)+H(x,y)\right]=\frac{1}{\beta }\ln p(x|y)+H(x,y) \end{array}$$

With these preliminaries in place, we are now able to define the problem for which mean-field theory is the solution. This problem arises when we know only the Hamiltonian. Simply put, the partition function (*Z*) is hard to compute. This is due to the difficulty of calculating the integral in Equation (1) for all but the simplest Hamiltonians. Without the partition function, we cannot calculate the conditional density of *x* given *y*. The mean-field approach starts by considering a simpler (reference) system, where there are no interactions between the constituents of the system. This absence of interactions is known as a mean-field assumption:

(4) $$\begin{array}{l} q(x|y)=\prod_{i}q(x_{i}|y)\\ q(x_{i}|y)=\frac{e^{-\beta h_{i}(x_{i},y)}}{Z_{i}(y)}\\ H_{q}(x,y)=\sum_{i}h_{i}(x_{i},y) \end{array}$$

This system factorises into a series of marginal distributions in virtue of the decomposition of the Hamiltonian into a sum of Hamiltonians for each component of the system. We refer to the distribution *q* as a variational density [19]. At this point, we can appeal to the Bogolyubov inequality [20]. This is a special case of Jensen’s inequality that says that the Helmholtz free energy of the interacting system is always less than if we calculated the free energy using the original Hamiltonian but replace the conditional probability with the variational density. We refer to the latter as the variational free energy and use the subscript *q* to distinguish this from the Helmholtz free energy. Re-expressing in terms of a Kullback-Leibler (KL) Divergence (A relative entropy (the average log ratio of two densities) that is always greater than or equal to zero (by Jensen’s inequality)) the Bogolyubov inequality becomes clear:

(5) $$\begin{array}{ll} F_{q}(y) & ≜E_{q(x|y)}\left[\frac{1}{\beta }\ln q(x|y)+H(x,y)\right]\\ & =E_{q(x|y)}\left[\frac{1}{\beta }\ln q(x|y)-\frac{1}{\beta }\ln p(x|y)\right]-\frac{1}{\beta }\ln Z(y)\\ & =\frac{1}{\beta }\underset{\geq 0}{\underset{︸}{D_{KL}\left[q(x|y)||p(x|y)\right]}}+F(y)\\ & \geq F(y) \end{array}$$

Equation (5) says that the variational free energy (*F_q_*) is an upper bound on the Helmholtz free energy (*F*). The implication is that, by minimising the latter, we should arrive at a good approximation of the former. This converts the difficult integration problem of Equation (1) into a much easier optimisation problem. Variational approaches of this sort have a long history, perhaps most famously in the formulation of quantum mechanics in terms of distributions over alternative paths a particle might follow [21]. Crucially, the factorisation of the variational density means we can optimise each factor independently. It is this property that lends a modular aspect to particular kinds of random dynamical system.

**Figure 1:**
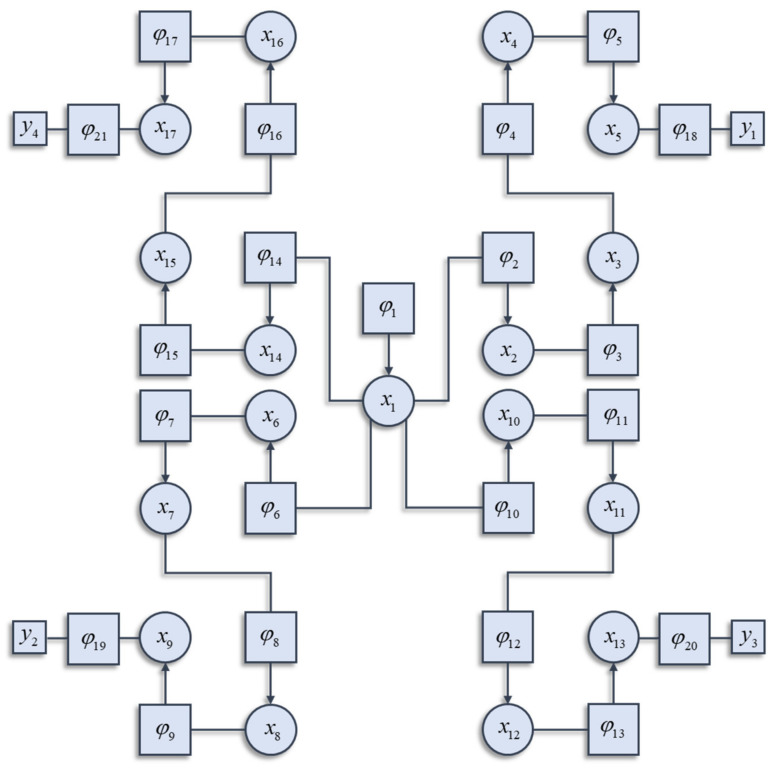
This schematic illustrates how the decomposition of a Hamiltonian into the sum of potentials may be represented graphically. This is a factor graph that represents each potential as a square node. The arguments of each potential are represented as circles connected to that square node. The *y* arguments of the potentials are represented as smaller squares. The arrows on some of the edges inherit from the interpretation of potentials as log conditional probabilities. If a random variable A is conditionally dependent on a variable B, the factor linking the two will include an arrow pointing towards A. The factor graph shown here is the (arbitrarily constructed) Hamiltonian that we will employ in the simulations in subsequent figures. This assumes a quadratic form for each potential. The details of these potentials are not important and could be replaced with any alternative quadratic functions. For readers interested in the precise formulation used in the simulations that follow, please see the Matlab routines referred to in the software note. In brief, each potential is centred upon a linear function of the mode of the neighbouring potential. An important feature of this structure is the sparsity of conditional dependencies. Each factor connects at most two variables. We assume $x_{i}\in {\mathbb{R}}^{2}$ in what follows. Uppercase subscripts are used to identify larger groups of *x* (i.e., $x_{K}\in {\mathbb{R}}^{\geq 2}$), corresponding to the argument of a given potential.

To understand how the different factors interact, it is worth highlighting that the Hamiltonian of the interacting system can itself be decomposed into a sum of factors. These will not be the independent factors of the mean-field reference system. Instead, they are conditional probability densities. Many elements in the sum are functions of more than one component of the system, and each component can contribute to more than one factor. Figure 1 illustrates a graphical notation used to represent the decomposition of a Hamiltonian. This general formalism has been exploited in signal processing [22], Newtonian [23] and quantum dynamics [24], and neurobiology [25,26]. Each square factor indicates a potential (*φ_K_*) whose argument (*x_K_*) is some subset of *x* from a region (*K*) of the graph involved in that potential. For example, region 6 includes (*x*_1_, *x*_6_), as these are the variables linked to the *φ*_6_ node. Crucially, regions overlap such that *x*_6_ participates in regions 6 and 7. The Hamiltonian is given by the sum of these potentials:

(6) $$H(x,y)=\sum_{K}\varphi_{K}(x_{K},y)$$

In general, not every potential will include *y* as an argument. In the example of Figure 1, only factors 18, 19, 20, and 21 include *y* as an argument, and each of these includes a different subset of the *y* variables. The Hamiltonian is constructed with three things in mind. The first is simplicity. To ensure this, we have used quadratic potentials that simplify the treatment of density dynamics in Section 3. The second is sparsity, which is a characteristic feature of brain-like systems. Sparsity means that each component of a system (e.g., neuron in a brain) interacts directly with relatively few other components. The third is that there are several different points at which the *y* variables may influence the system. This is consistent with alternative sensory modalities in nervous systems. We can now express the solution to the problem of finding the partition function as follows:

(7) $$\begin{array}{ll} q(x_{i}|y) & =\underset{q(x_{i}|y)}{\arg\min}F_{q}(y),\forall i\\ & \Leftrightarrow h_{i}(x_{i},y)=\sum_{\left\{K:x_{i}\in x_{K}\right\}}E_{q_{K\backslash i}}\left[\varphi_{K}(x_{K},y)\right]\\ & \Rightarrow F_{q}(y)\approx F(y)\\ & \Rightarrow q(x|y)\approx p(x|y)\\ q_{K\backslash i} & ≜\frac{q(x_{K}|y)}{q(x_{i}|y)} \end{array}$$

The approximate equality between the ‘*p*’ and ‘*q*’ distributions rests upon the assumption that the latter comprises a product of marginal factors (Equation (4))—which is not assumed for the former. The quality of the approximation may be quantified by the (negative) KL-Divergence between the two. Note that this is exactly the bound that appears in Equation (5) quantifying the difference between the associated partition functions. This accounts for the implication in Equation (7) that, when the partition functions are approximately equal, the KL-Divergence is approximately zero, and the ‘*p*’ and ‘*q*’ distributions are approximately equal. The second line expresses the ‘mean-field’—the average of the local potentials. There are two important things to draw from Equation (7). First, for the mean field associated with a factor, only the average values of the other factors matter. Second, we can ignore most of the terms in the sum of potentials comprising the original Hamiltonian. We only need those potentials in which our variable of interest participates, i.e., the ‘local’ potentials. This will become important in Section 5, where we revisit this idea in relation to the sparse connectivity structure of the brain [25].

While outside the scope of this paper, there are generalisations of mean-field theory that rely upon more sophisticated choices for the variational distribution. Cluster variational methods [27,28], based upon Kikuchi free energies, offer a much more general formulation. In brief, these employ a reference system with overlapping factors, corrected for the overlaps. It is the presence of these overlaps that distinguishes such approaches from mean-field theory, which is predicated upon the absence of overlaps. Table 2 sets out the form of the Hamiltonian associated with the variational distributions for a few key examples. Each of these is associated with a different inference scheme that minimises the associated variational free energy.

## 3. Non-Equilibrium Stochastic Dynamics

In this section, we take a step back and think about the dynamics of stochastic systems subject to the analyses of the previous section. These are systems that have attained a (possibly non-equilibrium) steady state, in the sense that the Hamiltonian is interpretable as a (static) log probability density. The first step in understanding what this means is to note that there are multiple equivalent ways in which the dynamics of a stochastic system may be formulated. We will focus upon two of these. One is a stochastic differential equation, which expresses equations of motion that depend upon a deterministic flow (*f*) and random fluctuations (*ω*). We will assume these fluctuations are normally distributed and uncorrelated over time or space. The second formulation we appeal to is afforded by a Fokker–Planck equation (a.k.a., a Kolmogorov forward equation). Instead of dealing with specific instances of a random system, Fokker–Planck equations deal with the dynamics of their probability density [30]. For concision throughout, we will use the dot notation to indicate partial time derivatives:

(8) $$\left.\begin{array}{l} \dot{x}=f(x,y)+\omega \\ E[\omega (\tau )\cdot \omega (t)]=2\Gamma \delta (\tau -t) \end{array}\right\}\Leftrightarrow \dot{p}(x|y)=\nabla_{x}\cdot \left(\left(\Gamma \nabla_{x}-f(x,y))p(x|y\right)\right)$$

In Equation (8), the amplitude of the random fluctuations is given by a diffusion tensor (2Γ). The δ-symbol indicates a Dirac delta function that ensures the covariance of the fluctuations at two time points (*t* and *τ*) is zero unless these times coincide, i.e., the fluctuations are temporally uncorrelated (c.f., a Wiener process). The Fokker–Planck equation on the right shows the rate at which probability mass enters or leaves an infinitesimally small region of space around *x*. Appendix A introduces the Fokker–Planck equation and links it to the stochastic differential equation on the left. However, the intuition is relatively simple. Imagine a drop of ink in water. Initially, the distribution of ink has a very sharp peak as it is concentrated in one place. This implies a large negative second derivative at this point, and relatively fast dispersion of the ink. As this peak is dispersed, and the second derivative becomes closer to zero, the rate at which ink leaves the initial location reduces. If the amplitude of fluctuations is greater (e.g., the water is boiling), the ink will spread out faster. This accounts for the term weighted by the diffusion tensor. The intuition for the role of the deterministic flow (*f*) is simpler. If there are currents in the water, the ink will leave those regions with fast flowing currents faster than regions of slower currents. The gradient of the current is key, as a positive gradient implies the currents leaving a region are faster than those entering it, while negative implies the opposite.

Using Equation (8), and the assumption that the rate of change of the probability density is zero when described by the Gibbs’ measure of Section 2, we can find an expression for the equations of motion in terms of the gradients of the Hamiltonian [31,32]:

(9) $$\begin{array}{l} p(x|y)\propto e^{-\beta H(x,y)}\Leftrightarrow \dot{p}(x|y)=0\\ \Rightarrow \nabla_{x}\cdot \left((\Gamma \nabla_{x}-f(x,y))e^{-\beta H(x,y)}\right)=0\\ \Rightarrow \Gamma \nabla_{x}e^{-\beta H(x,y)}-f(x,y)e^{-\beta H(x,y)}=Q\nabla_{x}e^{-\beta H(x,y)}\\ \Rightarrow f(x,y)=-\beta (\Gamma -Q)\nabla_{x}H(x,y) \end{array}$$

**Figure 2:**
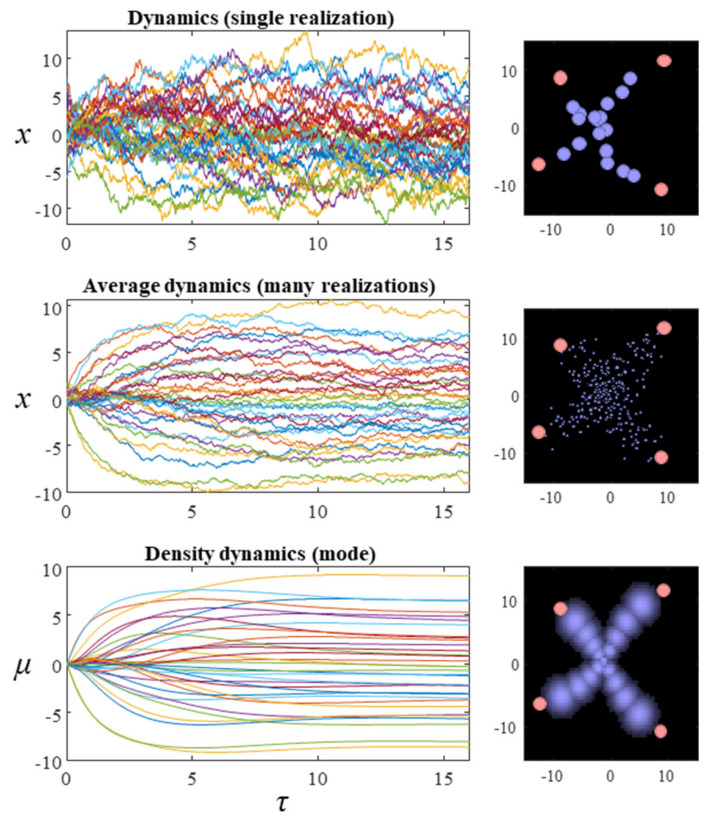
The plots in this figure illustrate the evolution of the random dynamical system whose Hamiltonian is shown in Figure 1. The plots on the left show the evolution of the system over time. This is a 34-dimensional system, which is shown on the right in terms of 17 particles, whose positions are described by two coordinates. The plots on the right show the final configuration at the end of the simulation. The first row shows a single realization of a stochastic trajectory. The second averages over 16 realizations of the trajectory. The third row shows the density dynamics under a Laplace approximation. The mean-field factorisation treats each particle independently (so each factor is a bivariate normal distribution). The filled pink circles in the plots on the right illustrate the values of the *y* variables (which are fixed). For ease of visibility, the intensity of each of the densities superimposed on this image have been normalised, such that their mode is the same intensity (regardless of the probability density at that mode).

The matrix *Q* is defined such that all its eigenvalues are pure imaginary or zero, ensuring the term on the right-hand side of the third line is divergence free. For the purposes of this paper, we will assume a block diagonal form for *Q*, where each matrix on the diagonal is a square, skew-symmetric matrix of dimension 2. We have assumed in the above that neither *Q* nor Γ vary with *x*. However, a more general form for Equation (9) can be constructed that allows these to vary [33]. The first and second rows of plots in Figure 2 show what happens when we simulate this system, by substituting the final line of Equation (9) into the stochastic differential equation in Equation (8). The temperature parameter (*β*) for these simulations is set at one. The dispersion of the steady-state density is therefore determined solely by the Hessian of the Hamiltonian. While simulating a single instantiation of these dynamics leads to a very noisy trajectory (first row of plots), simulating multiple instances and averaging reveals the self-organisation of this system into an ‘*x*’ shape.

While we could keep adding additional instances to this simulation and get incremental improvements to the characterisations of the distribution, a simple approach is to simulate the density dynamics directly. This gives us the results we would have found in the limit of infinitely many simulations of specific instances. The difficulty with this is that Fokker–Planck equations using the flow from Equation (9) directly involve an unwieldy covariance matrix for systems comprising many particles. However, we can simplify this problem by solving for individual factors. Applying the mean-field approximation from the previous section, we find the simpler expression:

(10) $$\begin{array}{l} q(x_{i}|y)\propto e^{-\beta h_{i}(x_{i},y)}\Leftrightarrow \dot{q}(x_{i}|y)=0\\ \Rightarrow f_{i}(x,y)=-\beta (\Gamma_{ii}-Q_{ii})\nabla_{x_{i}}h_{i}(x_{i},y) \end{array}$$

Note that this is only a function of *x_i_* and *y*, and no other system components. There is a sense in which this can be interpreted as ‘information encapsulation’ [34], one of the key features ascribed to modular architectures. Substituting this into the Fokker–Planck equation, we have:

(11) $$\dot{q}(x_{i}|y)=\nabla_{x_{i}}\cdot \left(\left(\Gamma_{ii}\nabla_{x_{i}}+\beta (\Gamma_{ii}-Q_{ii})\nabla_{x_{i}}h_{i}(x_{i},y))q(x_{i}|y\right)\right)$$

There are several methods available for solving Equation (11). Broadly, these include discretising over space or assuming a functional form for the probability density. For the former, this means integrating for each pixel (in two dimensions) based upon numerical gradients and Laplacians. The latter involves solving for the associated parameters. Either approach may be used here. We adopt the latter, which has the advantage of requiring fewer dimensions than a discretisation-based approach. Re-expressing this in terms of the sufficient statistics of the probability density—its mean and variance—we have (see Appendix B):

(12) $$\begin{array}{ll} {\dot{\mu }}_{i} & =-\beta \left(\Gamma_{ii}-Q_{ii}\right)E_{q(\mu_{i}|y)}\left[\nabla_{x_{i}}h_{i}\left(x_{i},y\right)\right]\\ & \approx -\beta \left(\Gamma_{ii}-Q_{ii}\right)\sum_{\left\{K:x_{i}\in x_{K}\right\}}\left({\left.\nabla_{x_{i}}\varphi_{K}\left(x_{K},y\right)\right|}_{x_{K}=0}+{\left.\nabla_{x_{i}x_{K}}\varphi_{K}\left(x_{K},y\right)\right|}_{x_{K}=0}\mu_{K}\right)\\ {\dot{\Sigma }}_{ii} & =2\Gamma_{ii}\\ & -\beta E_{q(x_{i}|y)}\left[\Delta x_{i}\nabla_{x_{i}}h_{i}{\left(x_{i},y\right)}^{T}\right]{\left(\Gamma_{ii}-Q_{ii}\right)}^{T}\\ & -\beta \left(\Gamma_{ii}-Q_{ii}\right)E_{q(x_{i}|y)}\left[\nabla_{x_{i}}h_{i}\left(x_{i},y\right)\Delta {x_{i}}^{T}\right]\\ & \approx 2\Gamma_{ii}\\ & -\beta \sum_{\left\{K:x_{i}\in x_{K}\right\}}{\left.\nabla_{x_{i}x_{i}}\varphi_{K}\left(x_{K},y\right)\right|}_{x_{K}=0}\Sigma_{ii}{\left(\Gamma_{ii}-Q_{ii}\right)}^{T}\\ & -\beta \left(\Gamma_{ii}-Q_{ii}\right)\sum_{\left\{K:x_{i}\in x_{K}\right\}}{\left.\nabla_{x_{i}x_{i}}\varphi_{K}\left(x_{K},y\right)\right|}_{x_{K}=0}\Sigma_{ii} \end{array}$$

The first of these equations sets out the dynamics of Equation (10)—the expected rate of change—under quadratic assumptions about the form of the Hamiltonian. For the simulations reported here, these assumptions hold by construction of the Hamiltonian as a quadratic function. More generally, this assumption depends upon local Taylor series approximations of the Hamiltonian. The second equation gives the dynamics of the covariance. Note that this equation is zero when the covariance is equal to the inverse of the sum of Hessian matrices (up to a scale factor *β*). The dynamics of the covariance provide an interesting perspective on the change in entropy of the system over time. Specifically, Equation (12) indicates that the rate of change of the covariance may be positive or negative. Remembering that the entropy of a normal distribution depends only on the covariance (and not the mode), we see that the system may increase or decrease its entropy. Consistent with the fluctuation theorems of stochastic thermodynamics [35], this highlights that the direction of change in entropy depends upon whether the initial or steady-state density has the greater dispersion. The third row of Figure 2 shows the results when Equation (12) is used to simulate the density dynamics of a system with the Hamiltonian of Figure 1. In Section 4, we unpack these dynamics in relation to modular theories.

Equation (12) can be simplified by introducing auxiliary variables (Π, *ε*):

(13) $$\begin{array}{l} {\dot{\mu }}_{i}\approx -\beta (\Gamma_{ii}-Q_{ii})\sum_{\left\{K:x_{i}\in x_{K}\right\}}\Pi_{i}^{K}\varepsilon_{i}^{K}\\ {\dot{\Sigma }}_{ii}\approx 2\Gamma_{ii}-\beta \sum_{\left\{K:x_{i}\in x_{K}\right\}}\Pi_{i}^{K}\Sigma_{ii}(\Gamma_{ii}-Q_{ii})T-\beta (\Gamma_{ii}-Q_{ii})\sum_{\left\{K:x_{i}\in x_{K}\right\}}\Pi_{i}^{K}\Sigma_{ii} \end{array}$$

The auxiliary variables are defined as follows:

(14) $$\begin{array}{l} \Pi_{i}^{K}≜{\left.\nabla_{x_{i}x_{i}}\varphi_{K}(x_{K},y)\right|}_{x_{K}=0}\\ \varepsilon_{i}^{K}≜\mu_{i}-\eta_{i}^{K}(\mu_{K\backslash i})\\ \eta_{i}^{K}(\mu_{K\backslash i})≜-{\left(\Pi_{i}^{K}\right)}^{-1}\left({\left.\nabla_{x_{i}x_{K\backslash i}}\varphi_{K}(x_{K},y)\right|}_{x_{K}=0}\mu_{K\backslash i}{\left.+\nabla_{x_{i}}\varphi_{K}(x_{K},y)\right|}_{x_{K}=0}\right) \end{array}$$

Equations (13) and (14) provide a useful intuition as to the behaviour of the system. It implies that the mode of each marginal density changes such that it minimises the difference (*ε*) between itself and a ‘target’ value (*η*), where the latter is a function of the modes of the other marginals with which it shares a potential. Each mode effectively chases (or ‘tracks’) a moving target until all modes have reached their attracting points. This mediates a form of synchronisation, on average, between the factorised components of the system. However, this does not mean the marginals contain information about the joint densities. Instead, interactions are mediated via the mean-fields as in Equation (7).

This treatment may sound very abstract and technical, however, it forms the basis for much of physics as we know it. Furthermore, it has enormous practical implications. Effectively, the simulations in Figure 2 show that it is possible to create highly structured ensemble dynamics (here a nonlinear 17-body problem with random fluctuations) with a desired ‘shape’. In other words, we can effectively write down a probabilistic description of what we want a system to look like, and then use the mean field approximation to realise that kind of system. In engineering, this would be known as directed self-assembly and is a central part of nanotechnology [36,37]. In the neurosciences, the (dynamic causal) modelling of neural interactions rests upon the mean field approximation in Equation (14) [38], creating a distinction between neural mass and mean field models [39].

The different perspectives on the same underlying dynamics shown in Figure 2 provide an interesting point of connection to different kinds of probabilistic inference scheme used widely in statistics and machine learning. Broadly, approximate inference techniques are divided into two classes. The first relies upon sampling, and include Markov Chain Monte Carlo (MCMC) approaches such as the Metropolis-Hastings algorithm [40] or Gibbs’ sampling [41,42]. Special cases of MCMC, including the Metropolis-adjusted Langevin algorithm [43] are based upon the dynamics given by Equation (9) to ensure a target distribution is attained after sufficient time. A more general form of Equation (9) has been used explicitly in constructing MCMC samplers [33]. The second approach is to work directly with the density dynamics by assuming a parameterised form for the density and optimising these parameters [19], i.e., variational inference. The first two rows of plots in Figure 2 can be thought of as showing how sampling approaches progress, while the third row is an example of a variational scheme. 

## 4. Factors and Modules

**Figure 3:**
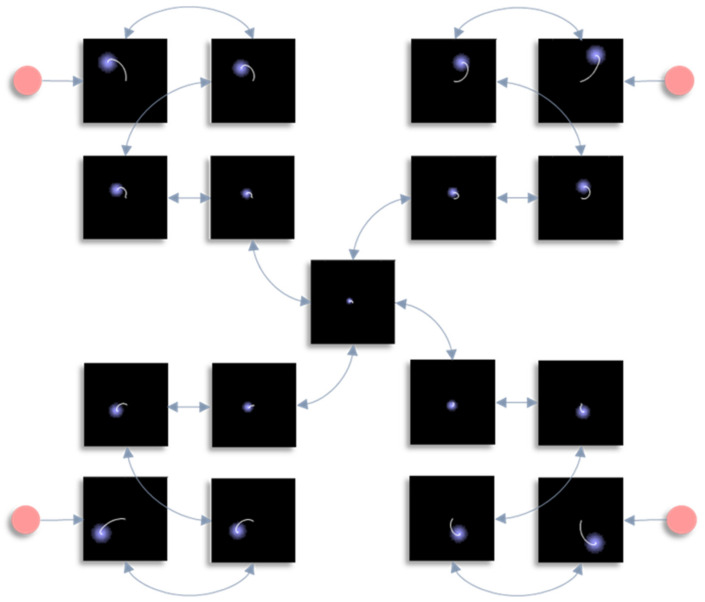
This figure decomposes the density dynamics of Figure 2 in line with the mean-field partition. The arrows here indicate the influence of each marginal density on another via their associated mean-fields. In other words, they represent the non-zero elements of the Jacobian for the vector *μ*, with elements *μ_i_*, whose rate of change is given in Equation (13). Each image shows the probability density at the end of the simulation (in blue) and the trajectory of the mode throughout the simulation (white). Note the highly precise distribution over the central factor, which is constrained by its four neighbours. The key message to take away from this figure is that the mean-field approximation separates the full system of Figure 2 into a series of smaller systems that influence one another only through their averages.

In this section, we draw upon the stochastic dynamics of Figure 2. The Hamiltonian that underwrites this specifies a pattern in which the location of each component of the system is conditionally dependent upon locations of other components. Our first step is to note that we can look at each of these components independently. Figure 3 shows the trajectory of the modes, and the final density, for each factorised density under the mean-field approximation. The reciprocal dependencies between these factors (i.e., the mean-fields) are shown as arrows. Note the spiral trajectories. These result from the combination of solenoidal and curl-free flows (down and around the gradients of the Hamiltonian, respectively). The decomposition of a single system into a series of interacting subsystems offers our first hint at ‘modularity’. 

**Figure 4:**
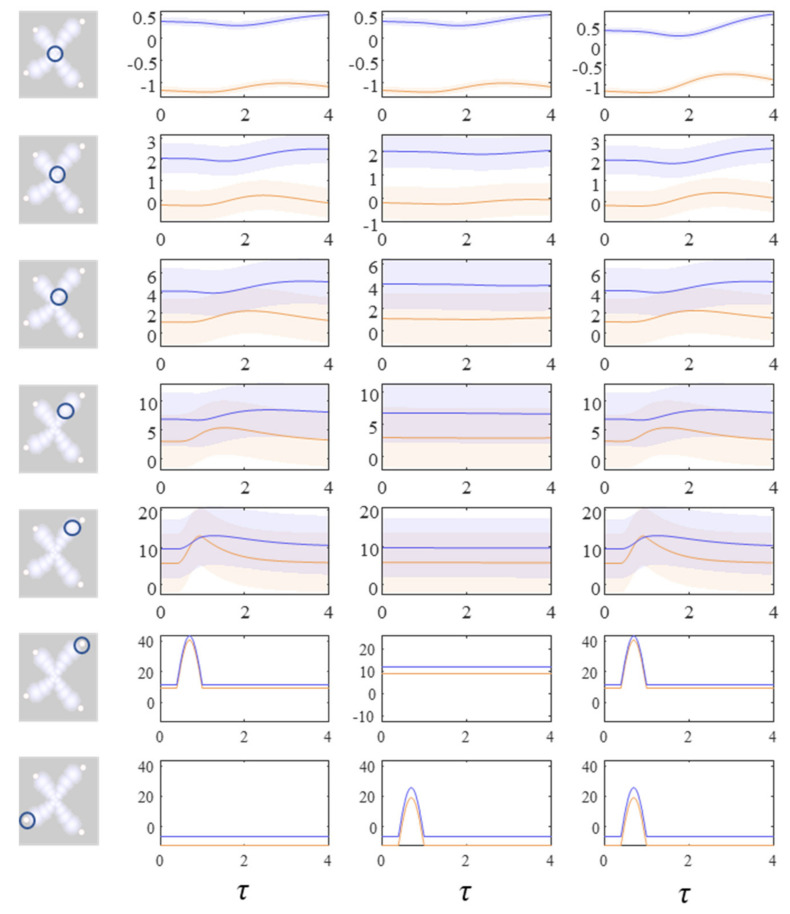
These plots show the consequences of perturbing the *y* variables on the density dynamics (depicted as the mode and surrounding 90% credible intervals) for each factor. The images on the left indicate which factor is shown in each row of the plots. Each column of plots shows a separate simulation in which different perturbations are applied to the *y* variables. In this figure, the plots show the central factor (first row) through to the upper right factor (fifth row). The lower two rows show the *y* variables in the upper right and lower left. These are perturbed by introducing a sinusoidal impulse. The first column of plots shows the response to the upper right perturbation. The second column shows the limited response to the lower left perturbation. The third column shows the increased recruitment of more central regions in the presence of both perturbations. The key point to take away from this figure is that a simple form of ‘information encapsulation’ or functional specialisation occurs in the extremities, with specific responses to, and only to, one sort of *y* variable. Over a hierarchy of timescales and progressively prolonged responses evocative of delay-period firing in working memory tasks, the factors become progressively multimodal. Figure 5 shows the lower left factors in the same simulation.

**Figure 5:**
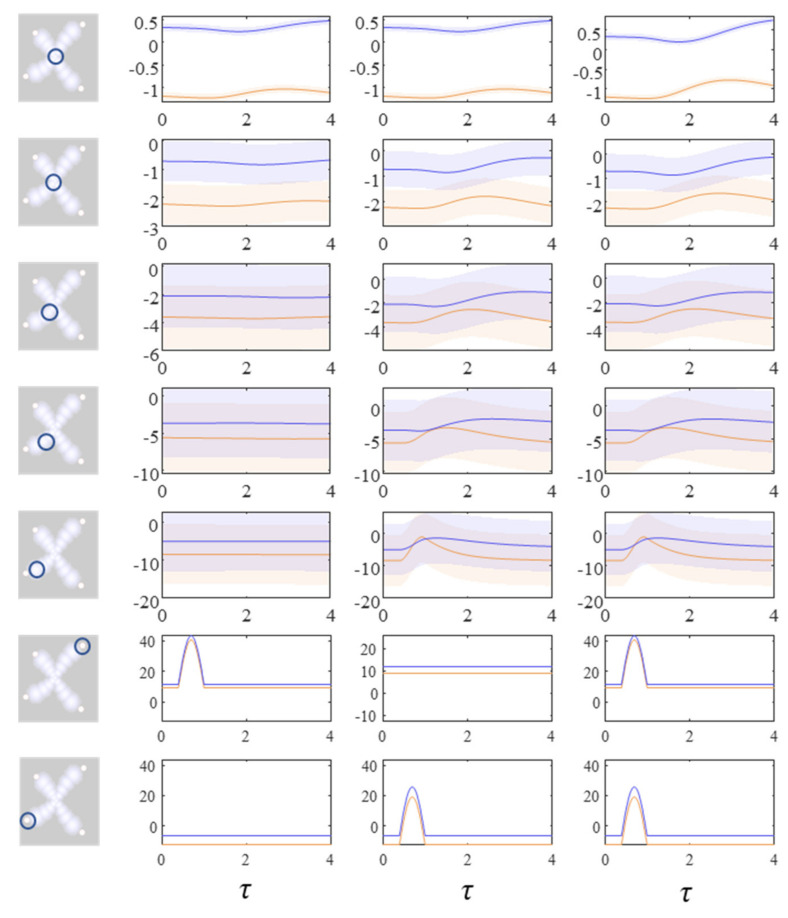
These plots complement those of Figure 4, illustrating the same perturbations and their consequences for the lower left modules. Here, there is little effect of the upper right *y* perturbation until we reach more central regions. However, there is a response to the lower left *y* perturbation that was not seen in Figure 4. For details on the format of these plots, please see the legend of Figure 4.

The next stage in our analysis is to think about the consequences of perturbing the system, to see how each marginal density responds. We can do this by interpreting *y* as sensory data and manipulating variables. This resembles standard approaches in neuroscience that measure the brain’s response to experimental sensory stimuli. Figure 4 and Figure 5 show what happens when we perturb the upper right (*y*_1_ in Figure 1) sensory input, the lower left (*y*_2_ in Figure 1) input, or both.

Figure 4 shows the density dynamics of the central and upper right factors (see Figure 3), while Figure 5 shows these for the central and lower left factors. There are three things to take away from these plots. First, they illustrate a form of functional specialisation, in that the lower left factors respond to changes in the lower left stimulus but not to the upper right stimulus, and vice versa for the upper right factors. In other words, we have segregated sensory streams that deal with different aspects of the sensorium: similar to cognitive processing associated with visual [44], auditory [45], language [46], and temporal [47] tasks. The second thing to note is that the timescale of the responses is slower (peaking later and persisting longer) the closer to the central factor (*x*_1_). This mimics the (slow and fast) temporal separation seen in neurobiological hierarchies [48,49,50,51]. It also implies a simple form of working memory, in the sense that the effects of the stimulus persist long after it has been removed. Finally, the more central factors respond to both sensory inputs, and show a greater response when both are presented simultaneously. Here, we have evidence in favour of multimodal factors analogous to those brain cells that respond to stimuli presented to different sensory modalities [52,53,54,55,56]. Multimodal properties of this sort speak to the importance of functional integration alongside modular segregation [2,57], heightened during cognitive processing [58].

## 5. Neuronal Message Passing

In the preceding sections, we used an arbitrarily constructed random dynamical system to illustrate a factorised (or modularised) account of systems with a sparse conditional independency structure at steady state. The resulting density dynamics show a form of functional segregation with distinct ‘sensory’ streams. As these converge upon one another, we see the emergence of a simple form of multisensory integration, based upon the expectation values of the sensory streams. Along these streams, each factor operates with a different temporal scale, much as sequences of cortical regions in sensory hierarchies. This illustrates that non-neural systems can behave as if they obeyed modular principles. In this section, we attempt to connect this back to the role of factorised dynamics in nervous systems.

**Figure 6:**
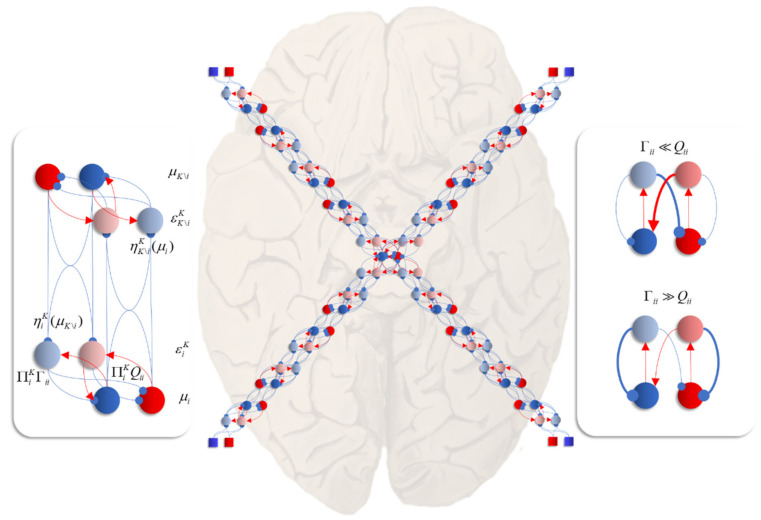
The central panel in this figure shows an interpretation of Equations (13) and (14), applied to the Hamiltonian of Figure 1, as a neuronal network. This shows a reciprocal message passing in which more central and more peripheral regions communicate along a neural hierarchy. Each arm of this hierarchy connects central regions to sensory input (shown as squares, consistent with previous figures). Central regions therefore have multimodal properties, responding to any of the sensory perturbations. Peripheral regions are more specialised in virtue of their proximity to external input. The panel on the left unpacks the connections between two regions (modules, or factors of a mean-field density) in detail. This includes neural populations representing the (2-dimensional) mode (in red and blue), auxiliary variables (in lighter shades) playing the role of prediction errors (i.e., gradients of the local Hamiltonian), and connections between these. Blue connections are inhibitory while red are connections excitatory. Note that, while some populations are shown as giving rise to both excitatory and inhibitory connections, we do not intend to imply a violation of Dale’s law. The assumption here is that there are intermediate inhibitory neurons that act to change the sign of the connection. The panel on the right highlights the importance of intrinsic (intra-modular) connectivity, and the role of the diffusion tensor (Γ) and solenoidal flow (*Q*) in determining neural activity. If the solenoidal component is large relative to the diffusion tensor, this leads to net excitation of the blue neuron by the red, and net inhibition of the red by the blue. This pattern of connectivity favours intrinsically driven oscillations. The circuit dominated by the diffusion tensor favours rapid convergence of neural activity to a fixed point.

The first point of contact is the role of local, reciprocal, interactions [59] as seen in the density dynamics. In this setting, a mean-field is essentially a description of the message passed to a given neuronal population. In a dynamical formulation, the gradients of the Hamiltonian potentials that comprise this mean-field are interpretable as synaptic weights. Figure 6 unpacks a neuronal network whose dynamics correspond to those above. The graphic on the left shows the interaction between expectations of a single factor and one of its neighbours that shares a local potential (i.e., a constituent of its Markov blanket [60]—the set of states that insulates a node from the rest of the network). Here, each factor may be thought of as predicting the other (via the *η* functions). This prediction is subtracted from the current expectation (*μ*) to give an error term (*ε*). The assumption here is that the time constants of the neural populations representing this error are very short relative to those of the expectations. The error term induces updates in the expectation such that it conforms to the prediction. This is a very simple (linear) form of predictive coding [61,62]—a prominent theory of brain function.

The central image shows what happens when there are multiple constituents to each Markov blanket. There are two ways in which this may manifest anatomically. The first, shown in each of the sensory streams, is that the error populations may accumulate predictions from each blanket constituent. The second is shown in the centre, where multisensory integration takes place. Here, there are multiple error terms, one from each constituent of the blanket. Intuitively, this is as if each error term gets a vote on the expectation, and the resulting attracting point is some combination of these. The influence of the error neurons on those populations representing expectations inherits the solenoidal and diffusion tensor terms. These may be interpreted as intrinsic (within-region) connectivity. The influence of these is shown in the graphic on the right of Figure 6. When the amplitude of fluctuations is large, the error neurons drive the expectations rapidly to their fixed point. However, when the solenoidal term is large, the reciprocal excitatory–inhibitory loop dominates, promoting oscillatory activity. Together, these terms contribute the damped oscillations that underwrite evoked response potentials in electrophysiological studies [63].

A second point of contact is that we have focused on the dynamics of conditional densities. The relevance of this is twofold. First, brain dynamics are generally studied by looking at neural responses to sensory stimuli (i.e., the neural dynamics conditioned upon sensation). Second, conditional densities of this kind underwrite the Bayesian brain hypothesis [64,65,66,67]. This view suggests that the brain employs a generative model comprising prior and likelihood densities to predict sensory data. This generative model is the Hamiltonian we have been discussing. 

Neural dynamics are then interpretable as forming posterior beliefs (conditioned upon data) about the causes of these data. In saying this, we have deliberately conflated two different perspectives on the Bayesian brain: we have interpreted our stochastic system as if it were a nervous system or a neural network. As such, the density dynamics reflect our beliefs, as observers, about that system, not the network’s beliefs about the outside world. In other words, the posterior is the probability of a neural state given an observation *y*. The other perspective is that, if we interpret the Hamiltonian as a generative model for *y*, the density dynamics acquire an interpretation as the brain’s inference about the causes of its sensory data. 

For the Hamiltonian used here, this implies some variable that has consequences for four different sensory modalities (*y*_1_,…,*y*_4_). For instance, the position of a cup of coffee has potential consequences for vision, gustation, olfaction, and somatosensation. It may be that the data-generating process is of a form that requires some transformation of the *x* variables, or even that the generative model is not an accurate description of the data-generating process [68]. Regardless of whether the model is a ‘good’ model, the inferential interpretation is useful in thinking about modularity. This is because it allows us to conceptualise a factor of the system as performing computations *about* something. If each factor is about something different, each can be thought of as a specialised module with a definitive role, in relation to the external environment.

In summary, we can interpret the dynamics of a system described by mean-field density dynamics in terms of messages (i.e., mean-fields) passed between module-like regions of a network [69,70,71]. For sufficiently sparse conditional dependency structures—like that of the Hamiltonian employed here—the message passing is evocative of synaptic communication in sparse neuronal networks. Interpreted as such, extrinsic (between-node) connection weights are determined by those terms in the Hamiltonian that contribute to a given mean-field. This is distinct to the intrinsic (within-node) connectivity. Intrinsic connections serve to optimise local potentials (given by summing the local mean-fields) through a combination of dissipative (gradient descent) and conservative (solenoidal) flows. Together these ensure a damped oscillation results during return to steady state following a perturbation. Finally, we highlighted the consistency of this perspective with Bayesian theories of brain function, interpreting conditional densities as posterior inferences about the causes of sensory data.

## 6. Discussion

The key message of this paper is that the concept of a ‘module’ simply refers to a factor of a probability distribution describing a system, and, implicitly, Bayesian beliefs held by a system. To underwrite this argument, we appealed to mean-field theory—a branch of statistical physics that deals with factorisation of probabilistic systems. We illustrated, using a system described by an arbitrarily constructed Hamiltonian, that the density dynamics of a high-dimensional stochastic system may be decomposed into factorised densities of low dimensional components that communicate with one another via their mean-fields. Finally, we interpreted this local communication in terms of synaptic message passing, highlighting the emergent distinction between intrinsic and extrinsic connectivity and the Bayesian interpretation of these dynamics. Crucially, this dynamical and inference architecture depends only on factorisation.

In the above, we have largely ignored the processes generating the variable *y*, which played the role of sensory data in the final section. While not necessary for the points we sought to address, including these processes has an important consequence for the way in which we think about the dynamics of sentient systems. Specifically, associating average flows of a system, subject to sensory perturbations, with average flows of the data-generating processes enables a reformulation of neuronal message passing in terms of the Hamiltonians of external dynamical systems. The Hamiltonian then becomes synonymous with a generative model of the data generating process. This Bayesian mechanical formulation [16] can be supplemented with the reciprocal influence, to account for neuronal influence on the external world (i.e., action). Things become even more interesting when we think about distributions over alternative trajectories of the internal, active, sensory, and external components of the system [72]. These give rise to the appearance of goal directed and exploratory behaviour. For introductions to the resulting active inference schemes, see [73,74].

We have kept things deliberately simple in the above, through use of a quadratic Hamiltonian. The treatment above, and in particular, the use of a Laplace assumption, retains validity in non-quadratic settings (e.g., [75,76]), but only in regions near the mode of the Hamiltonian. Clearly the assumption of a Gaussian variational density is inappropriate when the system tends towards multimodal densities. This is not a problem for the general principle of factorisation but does mean that solutions based upon the specific formulation of density dynamics used here are only locally valid. For a more general formulation, we could appeal to a more flexible family of variational distributions. An example would be a mixture of Gaussians (of the sort used in clustering applications). These allow for multimodal densities, through a linear combination of Gaussian densities with different modes [77]. In the setting of computational neuroscience, approaches of this sort have been employed to combine models of discrete decision making with those used to solve continuous inference problems [26]. Generative models of this sort have been used to simulate the interface between the selection and enaction of oculomotor saccades and [78], including the performance of oculomotor delay-period tasks [79] like those used in the study of working memory [80,81,82]. Such mixed models have also been used in the context of modelling neuroimaging data, to understand the way in which the brain switches between alternative connectivity states [83]. The implication here is that a more comprehensive understanding of the interaction between different factors of a neural system may require some factors representing Gaussian densities, and others categorical distributions over discrete variables.

Another interesting direction in which the formulations above may be extended is in tree decomposition of the Hamiltonians. This addresses the question of how certain kinds of mean-field assumptions (or more sophisticated approximations) may be justified by considering the structure of the Hamiltonian. An important idea here is that of tree-weighted re-parameterisation [84]. This is a class of methods designed to find alternative groupings (i.e., factorisations) of the variables in the graph describing the Hamiltonian. The idea is to create a simpler graph from the original by grouping together highly connected regions of the graph, while allowing for overlaps between groups. These methods provide an alternative perspective on the variational distributions in Table 2. Each Kikuchi approximation may be thought of as an alternative tree-weighted re-parametrisation. The utility of this perspective is that choices of alternative variational densities or trees may be scored. This scoring ends up approximating a KL-Divergence between the distributions under different parameterisations of the tree [85]—sharing the same fixed points as the associated free energy. As such, these techniques could be used to find the ‘best’ decomposition of a system. Another perspective on the same problem is that this decomposition rests upon finding a decomposition based upon Markov blankets in a dynamic setting. This uses adjacency matrices based upon a system Jacobian to find a decomposition such that each blanketed structure in the network is independent of all other structures given their blanket. For a numerical proof-of-principle of this approach, see [86].

Finally, it is worth considering what is gained by thinking about brain function in terms of interacting factors of a probability density. Ultimately, the gains are very similar to that of modularisation. From a neuroscientific point of view, to understand connectivity in the brain, it is necessary to know what the things being connected are [2]. In addition, it is useful to know that some aspects of brain function may be usefully studied in isolation, before placing this in the context of the wider neuronal network. More broadly, factorisation underwrites the notion of transfer learning or context invariance [87,88]. This is the idea that knowledge acquired in one context may be transferred over to a new scenario. Put simply, if we learn that water boils at a temperature of around 100°C, it should not matter if we change the context by moving to a new location. In the absence of transfer learning, this would have to be learned again in the new context. Each combination of location and temperature would be associated with its own belief about the likelihood of water having boiled. However, simply by factorising temperature and location, we can transfer our beliefs about the relationship between temperature and boiling to any location, c.f., carving nature at its joints via factorisation. Of course, moving to a location at a different altitude does change the temperature at which water boils. This is where the mean-field communication between factors becomes important, correcting for the drastic commitment to think of location and temperature as independent variables. While a trivial example, this highlights the fundamental relationship between factorisation and domain generality. The advantage of framing these problems explicitly in terms of mean-field theory, as opposed to modularity, is that it comes along with a well-established mathematical framework, whose legacy can be traced back to Occam’s principle [89] and the maximum entropy principle [90]. The simplicity of this perspective rests upon Equation (4) and the notion of factorisation. Both modular and mean-field accounts implicitly appeal to factorisations that enable descriptions of parts of a system (modules or marginals). The mean-field perspective is attractive because it does not require additional assumptions. It reformulates the challenge of understanding brain function to one of specifying the Hamiltonian (generative model) that the brain must solve and the variational distribution most appropriate for doing so. This sidesteps the anthropomorphised and *ad hoc* nature of modular accounts, in favour of a formalism grounded in the statistical physics of self-evidencing [91].

## 7. Conclusions

While not a definitive rejection of a modular perspective on brain function, the treatment presented here suggest that a simpler framing is in terms of factorisation and communication via mean-fields. The mean-field formulation preserves the notions of modular specialisation and information encapsulation. It allows us to work with probability densities within a factor of the variational distribution but does not require propagation of the full density between factors. This ensures a low dimensional passing of messages between factors, just as modules are thought to summarise the output of internal computations for the benefit of their neighbours. This provides a point of connection between Bayesian theories of brain function and the statistical message passing schemes thought to underwrite synaptic communication and computation. In short, the modular view of brain function may be the result of an intuitive application of mean-field theory. In making this explicit, we can draw upon developments in stochastic physics and develop a more formal, quantitative, account of neuronal organisation, from first principles.

## Figures and Tables

**Table 1:** Exponential family distributions.

Distribution	Support	Hamiltonian
Gaussian	x∈ℝ	$\frac{1}{2\beta }(x-\mu )\cdot \Pi (x-\mu )$
Multinomial ^1^	$\begin{array}{l} x_{i}\in \{0\ldots N\}\\ i\in \{1,\ldots ,K\}\\ \sum_{i}x_{i}=N \end{array}$	$-\frac{1}{\beta }\sum_{i}x_{i}\ln d_{i}$
Dirichlet ^2^	$\begin{array}{l} x_{i}\in (0,1)\\ i\in \{1,\ldots ,K\}\\ \sum_{i}x_{i}=1 \end{array}$	$\frac{1}{\beta }\sum_{i}(1-\alpha_{i})\ln x_{i}$
Gamma	x∈(0,∞)	$\frac{1}{\beta }\left(bx+(1-a)\ln x\right)$

^1^ Special cases include Categorical (*K* > 2, *N* = 1), Binomial (*K* = 2, *N* > 1), and Bernoulli (*K* = 2, *N* = 1) distributions. ^2^ A special case is the Beta distribution (*K* = 2).

**Table 2:** Variational distributions.

Name	Hamiltonian	Comments
Mean-field	$\sum_{i}h_{i}(x_{i},y)$	As in the main text, *x* is divided into non-overlapping subsets (*x_i_*), each of which is associated with its own Hamiltonian. The inference scheme associated with this approximation is known as *Variational message passing* [11].
Bethe	$\sum_{ij}h_{ij}^{\left(2\right)}(x_{i},x_{j},y)-\sum_{k}\left(c_{k}^{\left(1\right)}-1)h_{k}^{\left(1\right)}(x_{k},y\right)$	This expression uses a series of overlapping pairwise (superscript 2) Hamiltonians, that are then ‘corrected’ for these overlaps by subtracting singleton (superscript 1) Hamiltonians. Here, *c_k_* is the number of pairwise factors that include *x_k_* as an argument. The inference scheme associated with this approximation is known as *(loopy) Belief propagation* [29].
Kikuchi	$\begin{array}{l} \sum_{R}c_{R}^{\left(i\right)}h_{R}^{\left(i\right)}(x_{R}^{\left(i\right)},y)\\ c_{R}^{\left(i\right)}≜1-\sum_{\left\{K:R\subset K\right\}}c_{K}^{\left(i+1\right)} \end{array}$	This expression generalises the above approximations. Here, the subscripts index regions, while the superscript indexes the size of that region. In this expression, $x_{R}^{\left(i\right)}$ includes all elements of *x* in region *R* at scale *i*. Here, regions may overlap. If all regions are of size 1, this reduces to a mean-field approximation. If some are size 1 and others size 2, this is the Bethe approximation. Inference schemes based on the Kikuchi approximation are known as *Cluster variational methods* or *Generalised belief propagation* [27,28].

## Software Note

The simulations presented here may be reproduced and customised from Matlab (R2019a) code available at https://github.com/tejparr/Modules-or-Mean-Fields. Figure 2, Figure 3, Figure 4 and Figure 5 are generated by the DEMO_MeanFieldsModules.m script.

## Appendix A

This appendix provides a derivation of the Fokker–Planck Equation that lets us re-express the behaviour of a stochastic system in terms of its (deterministic) density dynamics. This is based upon the (more rigorous) treatment in [30] and is designed to provide some intuition as to the relationship between stochastic differential equations and their density dynamics. First, we note that the probability of *x* at time *τ* can be obtained by marginalising a joint density that includes this time and a previous time:

(A1) p(x,τ)=∫p(x,τ|x−Δx,τ−Δτ)p(x−Δx,τ−Δτ)dΔx

For the purposes of this appendix, we use the notation *p*(*x*, *τ*) to mean the probability density of *x* at time *τ*. We omit the conditioning on *y* that appears throughout the main text. Performing a Taylor series expansion (of *z* = *x* – Δ*x* around *x*) of the integrand, we can re-write this as:

(A2) $$\begin{array}{ll} p(x,\tau ) & =\int \sum_{n=0}\frac{1}{n!}{\left(-\Delta x\right)}^{n}{\left.\nabla_{z}^{n}p(z+\Delta x,\tau |z,\tau -\Delta \tau )p(z,\tau -\Delta \tau )\right|}_{z=x}d\Delta x\\ & =\sum_{n=0}\frac{{\left(-1\right)}^{n}}{n!}\nabla_{z}^{n}\int \Delta x^{n}{\left.p(z+\Delta x,\tau |z,\tau -\Delta \tau )d\Delta xp(z,\tau -\Delta \tau )\right|}_{z=x}\\ & =\sum_{n=0}\frac{{\left(-1\right)}^{n}}{n!}\nabla_{z}^{n}E_{p(z+\Delta x,\tau |z,\tau -\Delta \tau )}\left[\Delta x^{n}\right]{\left.p(z,\tau -\Delta \tau )\right|}_{z=x}\\ & =\sum_{n=0}\frac{{\left(-1\right)}^{n}}{n!}\nabla_{x}^{n}E_{p(\Delta x|\Delta \tau )}\left[\Delta x^{n}\right]p(x,\tau -\Delta \tau ) \end{array}$$

Subtracting the first term of the sum from both sides, we get:

(A3) $$p(x,\tau )-p(x,\tau -\Delta \tau )=\sum_{n=1}\frac{{\left(-1\right)}^{n}}{n!}\nabla_{x}^{n}E_{p(\Delta x|\Delta \tau )}\left[\Delta x^{n}\right]p(x,\tau -\Delta \tau )$$

From here, we can find the form of the rate of change of the probability density by taking limits:

(A4) $$\begin{array}{ll} \dot{p}(x,\tau ) & =\underset{\Delta \tau \to 0}{\lim}\left\{\frac{1}{\Delta \tau }\left(p(x,\tau )-p(x,\tau -\Delta \tau )\right)\right\}\\ & =\sum_{n=1}\frac{{\left(-1\right)}^{n}}{n!}\nabla_{x}^{n}\underset{\Delta \tau \to 0}{\lim}\left\{E_{p(\Delta x|\Delta \tau )}\left[\frac{\Delta x^{n}}{\Delta \tau }\right]\right\}p(x,\tau ) \end{array}$$

For the first two terms in the expansion, we have:

(A5) $$\begin{array}{ll} \lim_{\Delta \tau \to 0}\left\{E_{p(\Delta x|\Delta \tau )}\left[\frac{\Delta x}{\Delta \tau }\right]\right\} & =\lim_{\Delta \tau \to 0}\;\left\{E_{p(\Delta x|\Delta \tau )}\left[\frac{\int_{\tau }^{\tau +\Delta \tau }f(x,y)+\omega (t)dt}{\Delta \tau }\right]\right\}=f\left(x,y\right)\\ \lim_{\Delta \tau \to 0}\;\left\{E_{p(\Delta x|\Delta \tau )}\left[\frac{\Delta x^{2}}{\Delta \tau }\right]\right\} & =\underset{=0}{\underset{︸}{\lim_{\Delta \tau \to 0}\;\left\{E_{p(\Delta x|\Delta \tau )}\left[\frac{\int_{\tau }^{\tau +\Delta \tau }f(x,y)dt\int_{\tau }^{\tau +\Delta \tau }f(x,y)dt}{\Delta \tau }\right]\right\}}}\\ & +2\underset{=0}{\underset{︸}{\lim_{\Delta \tau \to 0}\;\left\{E_{p(\Delta x|\Delta \tau )}\left[\frac{\int_{\tau }^{\tau +\Delta \tau }f(x,y)dt\int_{\tau }^{\tau +\Delta \tau }\omega (t)dt}{\Delta \tau }\right]\right\}}}\\ & +\lim_{\Delta \tau \to 0}\;\left\{E_{p(\Delta x|\Delta \tau )}\left[\frac{\int_{\tau }^{\tau +\Delta \tau }\omega (t)dt\int_{\tau }^{\tau +\Delta \tau }\omega (t)dt}{\Delta \tau }\right]\right\}\\ & =\lim_{\Delta \tau \to 0}\;\left\{E_{p(\Delta x|\Delta \tau )}\left[\frac{\int_{\tau }^{\tau +\Delta \tau }\int_{\tau }^{\tau +\Delta \tau }\omega (t)\omega (s)dtds}{\Delta \tau }\right]\right\}\\ & =\lim_{\Delta \tau \to 0}\;\left\{\frac{\int_{\tau }^{\tau +\Delta \tau }\int_{\tau }^{\tau +\Delta \tau }2\Gamma \delta (t-s)dtds}{\Delta \tau }\right\}=2\Gamma \end{array}$$

As such, the density dynamics (up to the second order expansion) may be expressed as follows:

(A6) $$\dot{p}(x,\tau )=\nabla \cdot \left(\Gamma \nabla -f(x)\right)p(x,\tau )$$

This is the Fokker–Planck equation. Its utility is that, in place of studying specific instances of a stochastic trajectory, we can work with a deterministic equation that tells us how densities change over time. It may seem arbitrary to truncate the expansion in Equation A4 after the second term. The reason for doing so is that, by the Pawula theorem, additional terms support evolution to densities that are inconsistent with probability densities unless an infinite number of terms are included to preclude this.

## Appendix B

To use a Fokker–Planck formulation of dynamics practically, it is often necessary to find some parameterisation of the probability density such that the rate of change of the density may be reformulated in terms of the rate of change of the parameters of that density. One of the simplest options here is to take a Taylor series approximation to the log probability density. When this is truncated after the quadratic term, this is known as a Laplace approximation [38]. Here, we assume the log variational density is quadratic:

(A7) $$\begin{array}{l} \ln q(x_{i}|y)=\ln q(\mu_{i}|y)+(x_{i}-\mu_{i})\cdot \underset{=0}{\underset{︸}{{\left.\nabla_{x_{i}}\ln q(x_{i}|y)\right|}_{x_{i}=\mu_{i}}}}-\frac{1}{2}(x_{i}-\mu_{i})\cdot \Sigma_{ii}^{-1}(x_{i}-\mu_{i})\\ \Sigma_{ii}^{-1}≜-{\left.\nabla_{x_{i}x_{i}}\ln q(x_{i}|y)\right|}_{x_{i}=\mu_{i}}\\ \mu_{i}≜\underset{x_{i}}{\arg\max}\left\{q(x_{i}|y)\right\}\\ \Rightarrow q(x_{i}|y)=N(\mu_{i},\Sigma_{i}^{-1}) \end{array}$$

When dealing with linear dynamical systems, the Laplace approximation is exact. More generally, it is suitable for describing systems in the vicinity of the mode of the variational density. With this assumption in place, we can find expressions for the rate of change of the sufficient statistics of the probability density:

(A8) $$\begin{array}{ll} \mu_{i} & ≜E_{q(x_{i}|y)}[x_{i}]=\int_{-\infty }^{\infty }x_{i}q(x_{i}|y)dx_{i}\\ {\dot{\mu }}_{i} & =\int_{-\infty }^{\infty }x_{i}\dot{q}(x_{i}|y)dx_{i}\\ & =\int_{-\infty }^{\infty }x_{i}\nabla_{x_{i}}\cdot \left(\Gamma_{ii}\nabla_{x_{i}}q(x_{i}|y)\right)dx_{i}+\beta \int_{-\infty }^{\infty }x_{i}\nabla_{x_{i}}\cdot \left(\left(\Gamma_{ii}-Q_{ii})\nabla_{x_{i}}h_{i}(x_{i},y)q(x_{i}|y\right)\right)dx_{i} \end{array}$$

Integration by parts gives:

(A9) $$\begin{array}{l} {\dot{\mu }}_{i}=\underset{=0}{\underset{︸}{{\left[x_{i}\Gamma_{ii}\nabla_{x_{i}}q(x_{i}|y)\right]}_{-\infty }^{\infty }}}-\underset{=0}{\underset{︸}{\int_{-\infty }^{\infty }\Gamma_{ii}\nabla_{x_{i}}q(x_{i}|y)dx_{i}}}\\ \beta \underset{=0}{\underset{︸}{{\left[x_{i}(\Gamma_{ii}-Q_{ii})\nabla_{x_{i}}h_{i}(x_{i},y)q(x_{i}|y)\right]}_{-\infty }^{\infty }}}-\beta \int_{-\infty }^{\infty }(\Gamma_{ii}-Q_{ii})\nabla_{x_{i}}h_{i}(x_{i},y)q(x_{i}|y)dx\\ =-\beta (\Gamma_{ii}-Q_{ii})E_{q(x_{i}|y)}\left[\nabla_{x_{i}}h_{i}(x_{i},y)\right] \end{array}$$

The same procedure can then be applied to the covariance:

(A10) $$\begin{array}{ll} \Sigma_{ii} & ≜E_{q(x_{i}|y)}[\Delta x_{i}\Delta x_{i}^{T}]=\int_{-\infty }^{\infty }\Delta x_{i}\Delta x_{i}^{T}q(x_{i}|y)dx_{i}\\ {\dot{\Sigma }}_{ii} & =\int_{-\infty }^{\infty }\Delta x_{i}\Delta x_{i}^{T}\dot{q}(x_{i}|y)dx_{i}\\ & =\int_{-\infty }^{\infty }\Delta x_{i}\Delta x_{i}^{T}\nabla_{x_{i}}\cdot \left(\Gamma_{ii}\nabla_{x_{i}}q(x_{i}|y)\right)dx_{i}\\ & +\beta \int_{-\infty }^{\infty }\Delta x_{i}\Delta x_{i}{}^{T}\nabla_{x_{i}}\cdot \left(\left(\Gamma_{ii}-Q_{ii})\nabla_{x_{i}}h_{i}(x_{i},y)q(x_{i}|y\right)\right)dx_{i} \end{array}$$

Again, integrating by parts gives:

(A11) $$\begin{array}{ll} {\dot{\Sigma }}_{ii} & =\underset{=0}{\underset{︸}{{\left[\Delta x_{i}\Delta {x_{i}}^{T}\Gamma_{ii}\nabla_{x_{i}}q\left(x_{i}|y\right)\right]}_{-\infty }^{\infty }}}-\int_{-\infty }^{\infty }\left(\Delta x_{i}{\left(\Gamma_{ii}\nabla_{x_{i}}q\left(x_{i}|y\right)\right)}^{T}+\Gamma_{ii}\nabla_{x_{i}}q\left(x_{i}|y\right)\Delta {x_{i}}^{T}\right)dx_{i}\\ & +\beta \underset{=0}{\underset{︸}{{\left[\Delta x_{i}\Delta {x_{i}}^{T}\left(\Gamma_{ii}-Q_{ii}\right)\nabla_{x_{i}}h_{i}\left(x_{i},y\right)q\left(x_{i}|y\right)\right]}_{-\infty }^{\infty }}}\\ & -\beta \int_{-\infty }^{\infty }\left(\Delta x_{i}{\left(\left(\Gamma_{ii}-Q_{ii}\right)\nabla_{x_{i}}h_{i}\left(x_{i},y\right)\right)}^{T}+\left(\Gamma_{ii}-Q_{ii}\right)\nabla_{x_{i}}h_{i}\left(x_{i},y\right)\Delta {x_{i}}^{T}\right)q\left(x_{i}|y\right)dx_{i}\\ & =-2\Gamma_{ii}\underset{=0}{\underset{︸}{{\left[\Delta x_{i}q\left(x_{i}|y\right)\right]}_{-\infty }^{\infty }}}+2\Gamma_{ii}\underset{=1}{\underset{︸}{\int_{-\infty }^{\infty }q\left(x_{i}|y\right)dx_{i}}}\\ & -\beta E_{q(x_{i}|y)}\left[\left(\Delta x_{i}{\left(\left(\Gamma_{ii}-Q_{ii}\right)\nabla_{x_{i}}h_{i}\left(x_{i},y\right)\right)}^{T}+\left(\Gamma_{ii}-Q_{ii}\right)\nabla_{x_{i}}h_{i}\left(x_{i},y\right)\Delta {x_{i}}^{T}\right)\right]\\ & =2\Gamma_{ii}\\ & -\beta E_{q(x_{i}|y)}\left[\Delta x_{i}\nabla_{x_{i}}h_{i}{\left(x_{i},y\right)}^{T}\right]{\left(\Gamma_{ii}-Q_{ii}\right)}^{T}\\ & -\beta \left(\Gamma_{ii}-Q_{ii}\right)E_{q(x_{i}|y)}\left[\nabla_{x_{i}}h_{i}\left(x_{i},y\right)\Delta {x_{i}}^{T}\right] \end{array}$$

Together, Equations (A9) and (A11) provide parameterised forms for the density dynamics, and a tractable method of numerically integrating a Fokker–Planck Equation.
